# Partial Left Main Coronary Artery Obstruction Due to Migration of a Bioprosthetic Aortic Valve 60 Days After Transcatheter Aortic Valve Replacement: A Rare Clinical Case

**DOI:** 10.7759/cureus.93568

**Published:** 2025-09-30

**Authors:** Stefanos Votsis, Ioannis Tziatzios, Christos A Papanastasiou, Stavros Hadjimiltiades

**Affiliations:** 1 Cardiology, Medical College of Georgia at Augusta University, Augusta, USA; 2 Cardiology, 424 Military Hospital, Thessaloniki, GRC; 3 Cardiology, Aristotle University School of Medicine, Thessaloniki, GRC; 4 Cardiology, American Hellenic Educational Progressive Association (AHEPA) Hospital, Aristotle University of Thessaloniki, Thessaloniki, GRC

**Keywords:** complication of treatment, intraaortic balloon pump, late bioprosthesis migration, left main occlusion with cardiogenic shock, tavr (transcatheter aortic valve replacement)

## Abstract

Transcatheter aortic valve replacement (TAVR) offers a less invasive treatment alternative to surgical aortic valve replacement for high-risk patients. Although the procedure can be performed at low risk, life-threatening complications may arise in single cases during or even months after the procedure. Coronary obstruction has long been recognized as a potential complication of TAVR and is generally understood to occur within seconds or minutes after valve deployment. However, a few case reports have described the development of delayed coronary obstruction (DCO) occurring in the hours and days following the procedure. This is a case report of an 81-year-old patient who underwent TAVR due to aortic stenosis. The high implantation of a self-expandable valve, considered acceptable at the time of implantation, was eventually associated with severe myocardial ischemia that became clinically evident almost two months later. The patient underwent TAVR successfully, although the final aortogram revealed a somewhat high implant position. However, 50 days after the implantation, the patient started complaining of daily episodes of angina, dyspnea, and hypotension. She was admitted to the hospital 10 days later, and that day she developed a similar angina episode, followed by pulmonary edema and cardiogenic shock. The patient rapidly improved with intravenous vasoconstrictors and stabilized with the use of an intra-aortic balloon pump. A transthoracic echocardiogram (TTE) revealed a well-functioning prosthetic valve. An aortic root angiogram revealed patent coronary arteries. However, the non-coronary sinus of Valsalva (SOV) was barely opacified, and the left SOV was filling through a slit as a result of a prosthetic valve that had gradually migrated upwards in the sinotubular junction (STJ). This, in turn, resulted in compromised blood flow to the left main (LM) coronary artery ostium. The obstruction was gradually worsening over time, leading to myocardial ischemia and cardiogenic shock. An attempt was made to treat this condition interventionally by trying to cross into the left SOV and catheterize the LM ostium with the intention of percutaneous coronary intervention (PCI) treatment, but it was not successful. After these failed attempts, the decision was made to proceed with a bypass graft implantation to the left anterior descending artery, with off-pump surgery. Regrettably, the patient did not survive the operation.

## Introduction

Transcatheter aortic valve replacement (TAVR) is an effective therapy in patients with aortic stenosis. The most usual complications of this procedure are moderate/severe paravalvular leakage (PVL), major vascular and bleeding complications, disabling stroke, acute kidney injury (AKI), and conduction abnormalities, such as high-degree atrioventricular block with the necessity for permanent pacemaker implantation [1].

A less usual complication is coronary ostium occlusion, which is usually acute and occurs at the time of the valve implantation. Delayed onset coronary occlusion (DCO) following TAVR can occur weeks or even months after the procedure and presents a rare but clinically significant complication, which can be associated with a mortality rate as high as 50% [2]. It occurs in 0.22% of TAVR procedures, more commonly after valve-in-valve procedures (0.89% vs. 0.18% of native aortic valve procedures). In addition, DCO occurs more commonly if self-expanding valves are used during the index procedure as opposed to balloon-expandable devices (0.36% self-expandable vs. 0.11% balloon-expandable; p < 0.001) [2].

We report the case of a patient with severely calcified aortic valve stenosis, where the high implantation of a self-expandable valve, considered acceptable at the time of implantation, was eventually associated with severe myocardial ischemia that became clinically evident more than a month later.

## Case presentation

This is the case of an 81-year-old female patient with no history of arterial hypertension and no other risk factors for coronary artery disease, who developed dyspnea on exertion and peripheral edema over the past year, and episodes of pulmonary edema with persistent bilateral pleural effusions.

Following diuretic therapy, a transthoracic echocardiogram revealed a heavily calcified aortic valve (V_max_=5.71 m/sec and AV mean gradient of 68 mmHg with a calculated aortic valve area of 0.5 cm^2^), mild aortic regurgitation, moderate mitral regurgitation, and normal LV and RV systolic function with LV concentric hypertrophy (IVSDd=1.3 cm).

The patient's Euroscore (logistic) was 8.42%, the Euroscore II was 3.50%, and the Society of Thoracic Surgeons (STS) score for mortality was 2.466%. The patient was adamant about not having open heart surgery, and after a multislice CT evaluation of the valve and peripheral vessels, a decision was made to proceed with implantation of a self-expandable CoreValve ReValving system (Medtronic, Minneapolis, MN, USA).

**Figure 1:**
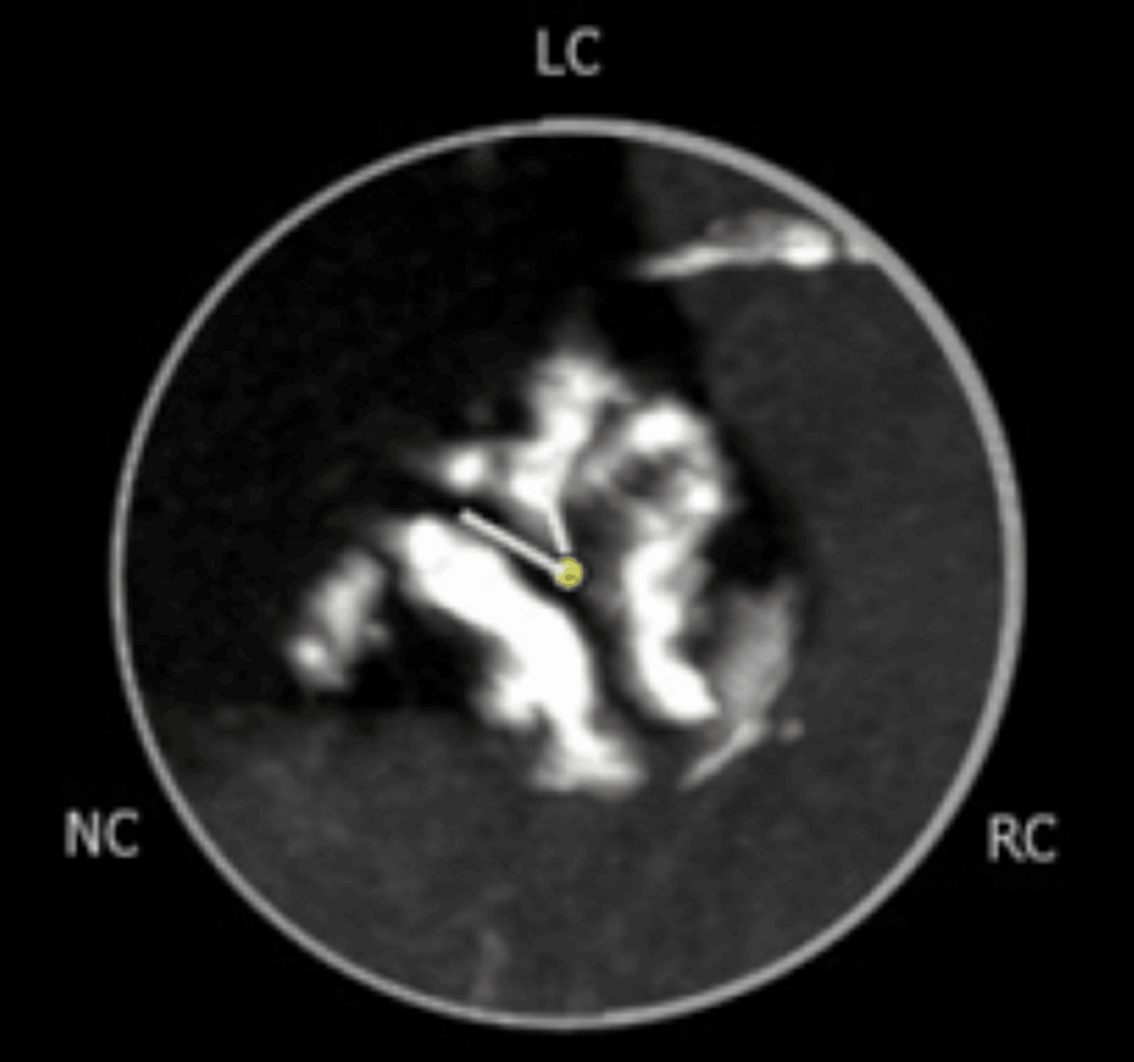
Cardiac CT image of the heavily calcified aortic valve before TAVR was performed. TAVR: transcatheter aortic valve replacement, LC: left cusp, NC: non-coronary cusp, RC: right cusp

The valve was heavily calcified (Figure 1) with a mean annulus diameter of 25.3 mm and a mean sinotubular junction (STJ) diameter of 26.5 mm. The left and right sinuses of Valsalva (SOV) had a height of 16.5 mm and 18.8 mm, respectively, and the diameters of the left coronary, right coronary, and noncoronary SOV were 31.5 mm, 30.2 mm, and 32.5 mm, respectively, all dimensions above the minimum required for a safe implantation. A coronary angiogram did not demonstrate any significant coronary artery disease.

**Figure 2:**
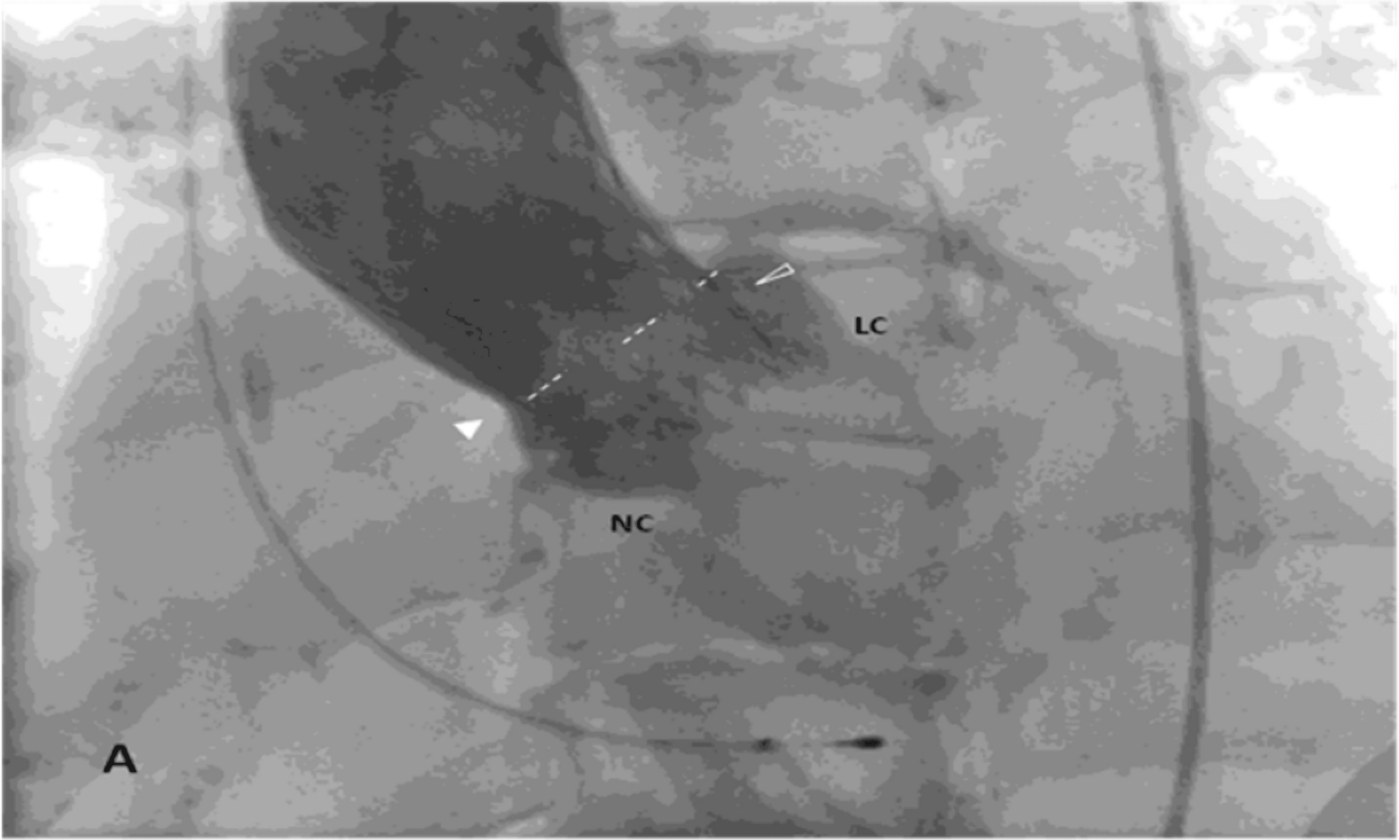
Aortogram immediately after the implantation of the CoreValve. All the SOV are opacified. The solid arrowhead points to the space between the wall of the STJ and the frame of the valve. The unfilled arrowhead points to the upper edge of the displaced left aortic cusp. The dashed line delimits the lowest edge of the upper part of the skirt. SOV: sinus of Valsalva, STJ: sinotubular junction

The implantation was performed under general anesthesia and transesophageal echocardiography (TTE) monitoring and evaluation. A balloon valvuloplasty was initially performed with a 22-mm Zmed II-X balloon (NuMED, Cross Roads, TX, USA), and a CoreValve 29 valve was implanted. Even before the final release of the valve, there was no significant paravalvular regurgitation and no restriction to flow to the coronaries, and the decision was made to release the valve despite the high implantation position. On closer inspection, it is evident that the lowest level of the upper edge of the skirt was close to the level of the STJ; the waist of the valve frame was higher than the level of the STJ, and there was a space of marginal width between the skirt of the valve and one side of the wall of the aorta at the level of the STJ (Figure 2).

The patient was discharged on aspirin and clopidogrel, clinically asymptomatic with no conduction abnormalities. Fifty days after the implantation, she started complaining of daily episodes of precordial pain on lying down, of 30 minutes' duration, usually after taking her antihypertensive medications. Consequently, she was scheduled for hospital admission 60 days after valve implantation for an inpatient diagnostic workout due to her symptoms.

On the day of her admission, she developed a similar episode of precordial chest pain with hypotension, this time followed by pulmonary edema and documented ECG ST depression in all leads except aVR and V1, where ST elevation was recorded. The patient rapidly improved with intravenous vasoconstrictors and stabilized with an intra-aortic balloon pump. A TTE revealed a well-functioning prosthetic valve.

**Figure 3:**
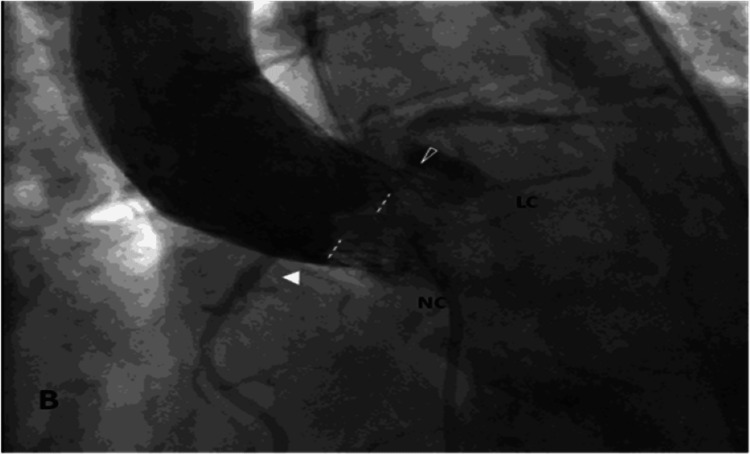
An aortogram late after the appearance of ischemia. An aortogram, in a more caudal projection, shows almost no opacification of the noncoronary (NC) and right SOV and the narrow communication to the left SOV (LC). The frame of the valve seems to be fully apposed to the wall at the STJ (solid arrowhead). The dashed line delimits the lowest edge of the upper part of the skirt.

An aortic root angiogram revealed patent coronary arteries. However, the non-coronary SOV was barely opacified, and the left SOV was filling through a slit (Figure 3) as a result of a prosthetic valve that had gradually migrated upwards in the STJ. This, in turn, resulted in compromised blood flow to the left main (LM) coronary artery ostium. The obstruction was gradually worsening over time, leading to myocardial ischemia and cardiogenic shock.

An attempt was made to treat this condition interventionally by obtaining right femoral arterial access and trying to cross into the left SOV and catheterize the LM ostium with a 6 Fr EBU 3.5 catheter with the intention of percutaneous coronary intervention (PCI) treatment, but it was not successful. Similarly unsuccessful were catheterization attempts with 6 Fr JL4, AL1, and AL2 guide catheters. After these failed attempts, the decision was made to proceed with a bypass graft implantation to the left anterior descending artery with off-pump surgery. Regrettably, the patient did not survive the operation. Table 1 presents the timeline of the case's progress.

**Table 1:** Timeline table of the case's progress TAVR: transcatheter aortic valve replacement, ECG: electrocardiogram, TTE: transesophageal echocardiography, IABP: intra-aortic balloon pump, SOV: sinus of Valsalva, CABG: coronary artery bypass graft

Case Timeline	
Day 0	Balloon valvuloplasty followed by TAVR.
Day 2	The patient was discharged from the hospital.
Day 50	Initiation of symptoms (angina, dyspnea, hypotension).
Day 60	Pre-scheduled hospital admission. Pulmonary edema with ST depression in the ECG. TTE reveals no bioprosthetic valve malfunction. Patient stabilization with vasoconstrictors and IABP. The cath lab aortogram reveals valve migration resulting in significant left SOV flow compromise. Failed left main PCI attempt.
Day 61	Unsuccessful CABG attempt (patient did not survive the operation).

## Discussion

Early (or acute) coronary obstruction after TAVR occurs between minutes and <7 days after valve implantation, whereas delayed (or late) coronary obstruction usually occurs >7 days after the procedure [2]. Late development of ischemia after TAVR with a CoreValve has also been reported [3] and has been associated with a high position of the implanted bioprosthesis; however, the mechanism underlying this association has not been elucidated. Explanted CoreValve valves have revealed the presence of thrombus and early intimal hyperplasia [4,5], which may compromise the flow to the sinuses, but the remodeling of the valve-annulus complex is a more plausible mechanism of reduced flow [6].

In our case, possible explanations for the occurrence of the late obstruction include thrombus formation, tighter sealing at the level of the STJ due to slow valve expansion or displacement, and, unlikely, fibrous tissue ingrowth, which is a late phenomenon. All of the above require that the space that permits the flow of blood between the skirt and the wall of the aorta, immediately post-implantation, be narrow enough to be easily compromised over time. Of note, our patient did not receive anticoagulation therapy immediately post-TAVR, as this is not considered a standard-of-care, and at the time, there was no suspicion of a thrombotic event.

One scenario of our case's late coronary obstruction is the slow, gradual migration of the valve frame from the place of initial implantation higher into the STJ and the ascending aorta. In all probability, this upward movement was facilitated by the already somewhat high (<5 mm below the annulus) valve position, which, although considered acceptable at the time (according to co-planar view fluoroscopic evaluation), proved unstable over time. The upward valve migration gradually brought the valve skirt into a new, higher position that had a "better" aortic wall apposition but that was opposite the LM ostium, causing its partial obstruction, which was worsening slowly over time. However, the aortogram at the time of the patient's second presentation does not manifest obstruction of the LM ostium (Figure 3).

Another, even more plausible scenario is that the upward valve migration did not directly obstruct the LM ostium; its skirt partially "sealed" the STJ, thus compromising blood flow to the aortic sinuses and, consequently, to the coronary arteries and especially the LM (Figure 2). This obstruction deterioration began by manifesting dyspnea and angina symptoms initially, leading to critical coronary ischemia resulting in cardiogenic shock later on. Unfortunately, transcatheter LM ostium engagement (already challenging due to the valve prosthesis struts and frame design) had a minimal chance of success because of the aforementioned lack of space between the valve frame, the valve skirt, and the LM ostium.

## Conclusions

Although extremely rare, post-TAVR delayed prosthetic valve migration is a potentially serious complication, as it can result in coronary ostium flow obstruction. Risk factors for this complication include (1) low coronary height, (2) small SOV diameter, (3) bulky leaflet calcification, (4) shallow STJ, and (5) high implant final position. Self-expandable valves and certain frames present specific access/interaction risks.

Our case demonstrates that (1) in the TAVR setting, further sealing at the STJ may occur as a late phenomenon with the CoreValve, so vigilance for delayed coronary flow obstruction is essential on the part of the medical team; (2) borderline high implants with narrow residual space at the STJ may prove unstable; (3) when the implantation is high and the unconstrained valve diameter is equal to or larger than the STJ diameter, the flow to the SOV may be compromised and ischemia may develop, even without direct obstruction of the coronary ostia; (4) in high-risk anatomy, the interventionalist may consider coronary protection/BASILICA; (5) meticulous planning of the procedure based on multimodality imaging is quintessential. CT scans can be helpful not only for valve sizing but also to decide on the depth of the valve deployment; and (6) early post-TAVR multimodality imaging is essential to diagnose potential caveats in a timely manner and thus mitigate the risk of late complications as much as possible.
